# Immunotherapy improves the prognosis of lung cancer: do we have to change intensive care unit admission and triage guidelines?

**DOI:** 10.1186/s13054-017-1602-8

**Published:** 2017-01-27

**Authors:** Antoine Guillon, Karen L. Reckamp, Nathalie Heuzé-Vourc’h

**Affiliations:** 1 CHRU de Tours, Service de Réanimation Polyvalente, F-37000 Tours, France; 2 INSERM, Centre d’Étude des Pathologies Respiratoires, U1100, F-37032 Tours, France; 3 Université François Rabelais de Tours, F-37032 Tours, France; 4 City of Hope Comprehensive Cancer Center, Duarte, CA USA

Bald heads may soon not be a sign that identifies a cancer patient receiving treatment. Indeed, therapies for cancer patients are improving dramatically leading to increased survival rates, and most are associated with a different toxicity profile. Recently, antibody-based therapy has transformed the therapeutic landscape and biology of non-small cell lung cancer (NSCLC) and other solid tumors. This may also reshuffle the playing cards for an intensive care unit (ICU) admission policy due to improved outcomes. In November 2016, the results of the KEYNOTE-024 trial showed for the first time the superiority of immunotherapy over chemotherapy as first-line treatment for NSCLC [1]. In this phase 3 trial, a humanized monoclonal antibody (mAb) against programmed death 1 (PD-1) was tested in patients who had previously untreated advanced NSCLC. The clinical trial was stopped by the safety monitoring committee on the basis of substantial clinical benefit of immunotherapy, and patients remaining in the chemotherapy group were switched to receive immunotherapy.

Immunotherapy with immune checkpoint inhibitors has ushered in a new chapter in the treatment of solid tumors. In place of directly targeting cancer cells, these drugs stimulate immune cells to enhance the host immune response against cancer. Two classes of immune checkpoint inhibitors with blocking antibodies have demonstrated a high level of antitumor activity in a variety of malignancies: (i) anti-cytotoxic T-lymphocyte antigen-4 antibodies (not yet approved in NSCLC); and (ii) inhibitors of PD-1 (nivolumab, atezolizumab, and pembrolizumab)—all established second-line therapeutic options for patients with metastatic NSCLC [2].

Antibody-based therapy in NSCLC is a therapeutic breakthrough—the first mAb was approved only a decade ago [3]. It is difficult to predict lung cancer mortality because new mAb therapeutic options continue to emerge and improve survival for patients. When a clinical trial is completed in lung cancer, the overall survival may change due to innovative immunotherapy, making previous results obsolete overnight. Indeed, the development of immunotherapy and combinations for lung cancer causes triage criteria for admission into the ICU to be in constant flux. We believe that we have entered a time when triage criteria based on lung cancer prognosis will be almost impossible to define for the ICU clinician. Consequently, ICU admissions will have to be determined by high-quality consultation between the intensivist and thoracic-oncologist to define prognosis and appropriate treatment goals. Intensivists should note that the prognosis and survival for lung cancer will be transformed by this mAb immunotherapy revolution.

## Abbreviations

ICU: Intensive care unit
mAb: Monoclonal antibody
NSCLC: Non-small cell lung cancer
PD-1: Programmed death 1
